# Modeling and Analyzing the Transmission Dynamics of HBV Epidemic in Xinjiang, China

**DOI:** 10.1371/journal.pone.0138765

**Published:** 2015-09-30

**Authors:** Tailei Zhang, Kai Wang, Xueliang Zhang

**Affiliations:** 1 School of Science/Chang’an University, Xi’an, China; 2 Department of Medical Engineering and Technology/ Xinjiang Medical University, Urumqi, China; Shanxi University, CHINA

## Abstract

Hepatitis B is an infectious disease caused by the hepatitis B virus (HBV) which affects livers. In this paper, we formulate a hepatitis B model to study the transmission dynamics of hepatitis B in Xinjiang, China. The epidemic model involves an exponential birth rate and vertical transmission. For a better understanding of HBV transmission dynamics, we analyze the dynamic behavior of the model. The modified reproductive number *σ* is obtained. When *σ* < 1, the disease-free equilibrium is locally asymptotically stable, when *σ* > 1, the disease-free equilibrium is unstable and the disease is uniformly persistent. In the simulation, parameters are chosen to fit public data in Xinjiang. The simulation indicates that the cumulated HBV infection number in Xinjiang will attain about 600,000 cases unless there are stronger or more effective control measures by the end of 2017. Sensitive analysis results show that enhancing the vaccination rate for newborns in Xinjiang is very effective to stop the transmission of HBV. Hence, we recommend that all infants in Xinjiang receive the hepatitis B vaccine as soon as possible after birth.

## Introduction

Hepatitis B is a potentially life-threatening liver infection caused by the hepatitis B virus (HBV). It is a major global health problem. It can cause chronic liver disease and chronic infection and puts people at high risk of death from cirrhosis of the liver and liver cancer. More than 240 million people have chronic (long-term) liver infections. More than 780, 000 people die every year due to the acute or chronic consequences of hepatitis B [1]. It is one of the top five infectious diseases in mainland China: around 130 million people are carriers of HBV, almost a third of the people infected by HBV worldwide, 30 million people are chronically infected, 300,000 people die from HBV-related diseases every year, accounting for 40–50% of HBV-related deaths worldwide [2]. According to hepatitis B data reported by the Ministry of Health of China from Jan. 2012-Dec. 2012, the new cases is 1,087,086 which is the top one among all infectious disease.

Xinjiang Uyghur Autonomous Region is an autonomous region of China in the northwest of the country. It is the largest Chinese administrative division. Hepatitis B is also one of major public health problems in Xinjiang which has been threatening the people in Xinjiang. At the end of 2012, the cumulative number of hepatitis B reported was approximately 350,000. In the paper [3], the authors explored the epidemic characteristics of different types of viral hepatitis in mainland China in 2011. The results shows Xinjiang belongs to high-prevalence region for HBV disease. Another paper [4] indicates that the HBV prevalence rate increased from 2.578‰ in 2005 to 5.0506‰ in Kashi prefecture, Xinjiang in 2012. The prevalence rate of viral hepatitis in Changji prefecture of Xinjiang is about 5.3648 ‰ in 2013, while HBV accounts for 85.77% of the total [5]. The situation of HBV transmission is very complicated and grim. Now, there are great potential of HBV transmission among the total population in Xinjiang.

Mathematical models have been used extensively in researching into not only the dynamical systems in spatial effects [6–10] but also the epidemiology of HBV disease to improve our understanding of the major contributing factors to the pandemic [2, 11–13]. Anderson and May used a simple mathematical model to illustrate the effects of carriers on the transmission of HBV [11]. An HBV transmission model was developed to explore the impact of vaccination and other controlling measures for HBV infection. The results shows that booster doses of hepatitis B vaccine are very necessary [14]. Khan et al. presented characteristics of HBV disease transmission in the form of a mathematical model. The effect of immigrants is analyzed in the model to study the effect of immigrants for the host population [15]. Zou et al. proposed a mathematical model to understand the transmission dynamics and prevalence of HBV in mainland China [2]. Furthermore, mathematical models have been used to analyze the dynamics of viral infections [16–18]. Pang et al. studied the dynamical behavior of a hepatitis B virus model with CTL immune responses. Mathematical analysis and numerical simulations show that the CTL immune responses play a significant and decisive role in eradication of disease [16].

Motivated by work mentioned above, we use an infectious disease model to understand the transmission dynamics and prevalence of HBV in Xinjiang, China. The paper is organized as follows. In the next section, we present the model formulation and the threshold value. In Section 3, we give the dynamical behavior of the model including, equilibria, stabilities and persistence. The parameter estimation and projection of HBV in Xinjiang are carried out in Section 4. In Section 5, we give a brief summary and some discussions.

## Materials and Methods

### Model Desciptions

**Fig 1:**
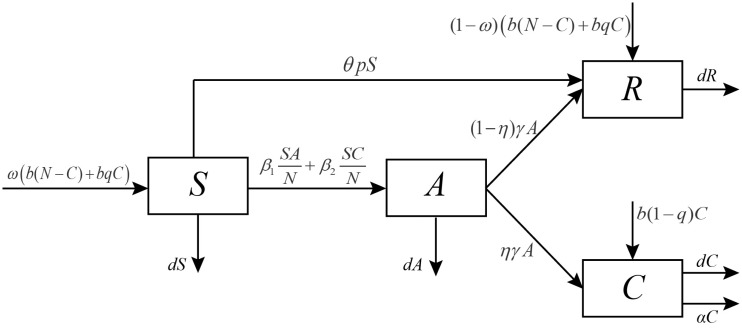
Schematic diagram of HBV transmission, structured. *S*(*t*), *A*(*t*), *C*(*t*) and *R*(*t*) represent susceptible, acute infection, chronic HBV carrier and recovered individuals, respectively.

We use a mathematical model to study the transmission of hepatitis B in Xinjiang, China. The total population is divided into four classes: the susceptible individuals *S*, acute infection individuals *A*, chronic HBV carriers *C*, and recovered individuals *R*. The total population is *N* = *S*+*A*+*C*+*R*. According to the natural history of HBV, we construct our model which is different from the previous HBV models which are mentioned in Introduction. Firstly, the birth rate function of the total population is not a constant. Secondly, we omit the rate of waning vaccine-induced immunity of vaccinated individuals, because there is no evidence to support the need for a booster dose of hepatitis B vaccine. Protection lasts at least 20 years, and is possibly life-long [19]. A flow diagram is depicted in Fig 1. The model is a system of four ordinary differential equations:

$$\{\begin{array}{ll} \frac{\text{d}S}{\text{d}t}= & \omega \left[b\left(N-C\right)+bqC\right]-\beta_{1}\frac{SA}{N}-\beta_{2}\frac{SC}{N}-\theta pS-dS,\\ \frac{\text{d}A}{\text{d}t}= & \beta_{1}\frac{SA}{N}+\beta_{2}\frac{SC}{N}-\left(d+\gamma \right)A,\\ \frac{\text{d}C}{\text{d}t}= & b\left(1-q\right)C+\eta \gamma A-\left(d+\alpha \right)C,\\ \frac{\text{d}R}{\text{d}t}= & \left(1-\omega \right)\left[b\left(N-C\right)+bqC\right]+\theta pS+\left(1-\eta \right)\gamma A-dR. \end{array}$$ (1)

The underlying assumptions of are listed as follows.

- The birth rate *b* is a constant and *bN* denotes newborns for total population.
- Horizontal transmission of infection may be expressed by standard incidence $\beta_{1}\frac{SA}{N}+\beta_{2}\frac{SC}{N}$, where *β*_1_ and *β*_2_ are the transmission coefficients of acute infection individuals *A* and chronic HBV carriers *C*, respectively.
- The total offspring of chronic HBV carriers *bC*, a fraction *q* of them are susceptible and a fraction 1 − *q* of them are infected at birth.
- *ω* is the proportion of newborns that is unsuccessfully immunized. Therefore, there is (1 − *ω*)(*b*(*N* − *C*)+*bqC*) within susceptible newborns who move directly to the recovered class.
- A proportion *p* of susceptible individuals are vaccinated and *θ* is the proportion of successful immunization.
- *d* is the natural mortality rate.
- *α* is the death rate induced by disease and we omit it for the acute infective.
- *γ* is the rate at which individuals leave the acute infection class. *η* is the proportion of leaving acute infection and progressing to carrier.

### Reproduction number and dynamical behaviors

The total population *N*(*t*) can be determined by the following differential equation,

$$\frac{dN}{dt}=\left(b-d\right)N-\alpha C$$ (2)

which is derived by adding the equations in Eq (1). Let $x=\frac{S}{N}$, $y=\frac{A}{N}$, $z=\frac{C}{N}$ and $r=\frac{R}{N}$ denote the fractions of the classes *S*, *A*, *C* and *R* in the total population, respectively. By definition,

x+y+z+r=1. (3)

From a direct computation using Eqs (1) and (3), we obtain the equations

$$\{\begin{array}{ll} \frac{\text{d}x}{\text{d}t}= & \omega \left[b\left(1-z\right)+bqz\right]-\beta_{1}xy-\beta_{2}xz-\left(b+\theta p\right)x+\alpha xz,\\ \frac{\text{d}y}{\text{d}t}= & \beta_{1}xy+\beta_{2}xz-\left(b+\gamma \right)y+\alpha yz,\\ \frac{\text{d}z}{\text{d}t}= & \eta \gamma y-\left(bq+\alpha \right)z+\alpha z^{2},\\ \frac{\text{d}r}{\text{d}t}= & \left(1-\omega \right)\left[b\left(1-z\right)+bqz\right]+\theta px+\left(1-\eta \right)\gamma y-br+\alpha rz. \end{array}$$ (4)

Since the variable *r* does not appear in the first three equations of system (4), we only need to study the first three equations of the system (4), thereby lowering the order of the system to be studied, i.e.,

$$\{\begin{array}{ll} \frac{\text{d}x}{\text{d}t}= & \omega \left[b\left(1-z\right)+bqz\right]-\beta_{1}xy-\beta_{2}xz-\left(b+\theta p\right)x+\alpha xz,\\ \frac{\text{d}y}{\text{d}t}= & \beta_{1}xy+\beta_{2}xz-\left(b+\gamma \right)y+\alpha yz,\\ \frac{\text{d}z}{\text{d}t}= & \eta \gamma y-\left(bq+\alpha \right)z+\alpha z^{2}. \end{array}$$ (5)

We determine *r* from *r* = 1 − *x* − *y* − *z*. From biological considerations, we study Eq (5) in the closed set

$$\Gamma =\{\left(x,y,z\right)\in R_{+}^{3}:0\leq x+y+z\leq 1\}.$$ (6)

where $R_{+}^{3}$ denotes the non-negative cone of ℝ^3^. It is clear that Γ is a positive invariant set with respect to Eq (5). System (5) always has a unique disease-free equilibrium $P_{0}\left(\bar{x},0,0\right)$, where

$$\bar{x}=\frac{\omega b}{b+\theta p}.$$

By using the concepts of next generation matrix and reproduction number presented in [20, 21], we compute the reproduction number of Eq (5). It can be called the modified reproductive number of the origin system (1). First, we change the order of system (5)

$${\left(\frac{dy}{dt},\frac{dz}{dt},\frac{dx}{dt}\right)}^{T}=F-V,$$

where

$$F=(\begin{array}{c} \beta_{1}xy+\beta_{2}xz\\ 0\\ 0 \end{array})$$

and

$$V=(\begin{array}{c} (b+\gamma )y-\alpha yz\\ -\eta \gamma y+\left(bq+\alpha \right)z-\alpha z^{2}\\ -\omega \left[b\left(1-z\right)+bqz\right]+\beta_{1}xy+\beta_{2}xz+\left(b+\theta p\right)S-\alpha xz \end{array}).$$

Then

$$F=(\begin{array}{cc} \beta_{1}\bar{x} & \beta_{2}\bar{x}\\ 0 & 0 \end{array}),V=(\begin{array}{cc} b+\gamma & 0\\ -\eta \gamma & bq+\alpha \end{array}).$$

*V* ^−1^ is given by

$$V^{-1}=\frac{1}{\left(b+\gamma )(bq+\alpha \right)}(\begin{array}{cc} bq+\alpha & 0\\ \eta \gamma & b+\gamma \end{array}).$$

Thus,

$$FV^{-1}=(\begin{array}{cc} \frac{\left[\beta_{1}\left(bq+\alpha \right)+\beta_{2}\eta \gamma \right]\bar{x}}{\left(b+\gamma )(bq+\alpha \right)} & \frac{\beta_{2}\bar{x}\left(b+\gamma \right)}{\left(b+\gamma )(bq+\alpha \right)}\\ 0 & 0 \end{array}).$$

The modified reproductive number is above matrix’s spectral radius, i.e.

$$\sigma =\frac{\left[\beta_{1}\left(bq+\alpha \right)+\beta_{2}\eta \gamma \right]\bar{x}}{\left(b+\gamma )(bq+\alpha \right)}.$$

For the stability of the disease-free equilibrium *P*_0_ for system (5). We first discuss its local stability as follows.

**Theorem 1.** *The disease-free equilibrium P_0_(x,0,0) of* Eq (5) *is locally asymptotically stable in Γ if σ < 1; it is unstable if σ > 1.*

We also have the following result on global stability of the disease-free equilibrium.

**Theorem 2.** *If σ_0_ < 1, then the disease-free equilibrium P_0_ is globally asymptotically stable, where*

$$\sigma_{0}=\frac{\beta_{1}\left(bq+\alpha \right)+\max\left\{\beta_{2},\alpha \right\}\eta \gamma }{\left(b+\gamma )(bq+\alpha \right)}.$$ (7)

Next, we show that the disease persists when *σ* > 1. We say the disease is endemic if the infected fraction (including acute and chronic stage) of the population persists above a certain positive level for large sufficiently time. That is to say there exists a *c* > 0 such that

$$\underset{t\to \infty }{lim inf}\left(y\left(t\right)+z\left(t\right)\right)\geq c.$$ (8)

The following result shows the disease will become an endemic under the meaning of persistence for the disease.

**Theorem 3.** *The disease of system* (5) *is uniform persistence in Γ if σ > 1.*

The proofs of Theorem 1, Theorem 2 and Theorem 3 are given in S1 Supporting Information (see S1 Supporting Information). From Theorem 1 together with Theorem 3, we can claim that the modified reproductive number *σ* is a threshold parameter which determines the outcome of disease. In other words, if *σ* < 1, the disease-free equilibrium *P*_0_ is asymptotically stable so that the disease dies out. While *σ* > 1, the disease will not go to extinction.

### Estimation of epidemiological parameters

**Fig 2:**
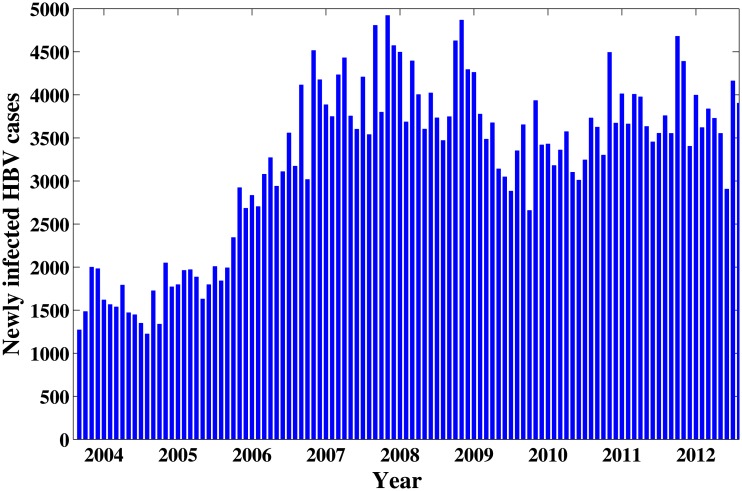
Monthly new reported HBV case in Xinjinag from 2004 to 2012. The Data was obtained from the website of public health science data center.

The model is applied to investigate the HBV infection in Xinjiang. We firstly need to estimate the model parameters in order to carry out the numerical simulations. The monthly new reported HBV cases in Xinjiang from January 2004 to December 2012 are obtained mainly from Public health science data center [22](see Fig 2).

Model (1) is considered as full model for our HBV fitness experiment. To test which biological assumption is more plausible to fit the HBV data, we need to employ model selection methods to evaluate different models [23]. Standard model selection criteria include Akaike information criterion (AIC) [24] and Bayesian information criterion(BIC) [25], and their variations such as AICc. Under a likelihood framework, these criteria can be written as

$$\text{AIC}=n\;\text{ln}\left(\frac{\text{RSS}}{n}\right)+2K,\;\text{BIC}=-2\;\text{ln}\;L+K\;\text{ln}\;n,$$

$$\text{AICc}=n\;\text{ln}\left(\frac{\text{RSS}}{n}\right)+\frac{2nK}{n-K-1},$$

where *L* is the likelihood function, *K* the number of parameters, *n* the sample size and RSS the residual sum of squares. We list submodels and these AIC, BIC, and AICc values in Table 1. The smaller AIC, BIC, or AICc value corresponds to a better model. From Table 1, we can see that these criteria select submodel 1 as the best model. Thus, our following discussion and data analysis will focus on submodel 1(*β*_1_ = 0, *θp* = 0).

**Table 1:** Model selection for HBV data.

Submodel	Assumptions	AIC	BIC	AICc
1	*β*_1_ = 0, *θp* = 0	111.9777	111.7695	112.9777
2	*β*_1_ ≠ 0, *θp* = 0	113.6415	117.6415	113.2250
3	*β*_1_ = 0, *θp* ≠ 0	124.9993	128.9993	124.5828

The values of parameters are listed in Table 2. We explain the parameter values as follows.

**Fig 3:**
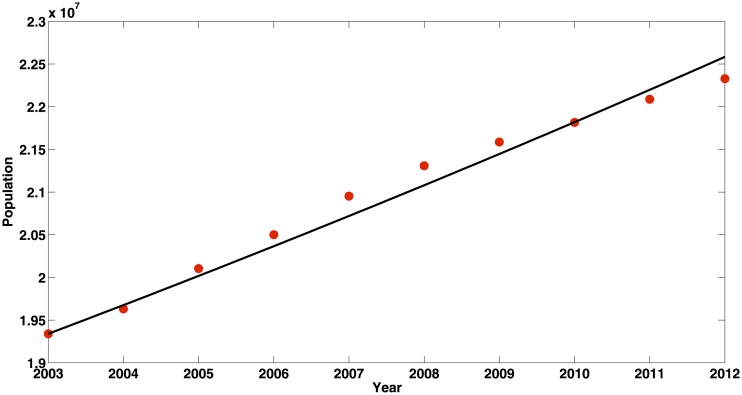
The number of population in Xinjiang from 2003 to 2012. The solid line is the fitted curve of total population number in Xinjiang.

- The average life expectancy of people in Xinjiang Uygur Autonomous Region of China was 71.12 years in 2005 [26]. We take it as the current average life expectancy. Thus, *d* = 1/71.12 = 0.0141. The demographic data from 2004 to 2012 for Xinjiang is listed in Table 3. We can obtain the annual birth rates is *b* = 0.0313 by least-square estimation(see Fig 3).
- From [12] and [2], we can obtain the parameters *ω*, *q*, *η*, *γ* and *α*(see Table 2).
- We define *M*(*t*) as the cumulative number of acute HBV case. Then, we have $M^{\prime }\left(t\right)=\beta_{1}\frac{SA}{N}+\beta_{2}\frac{SC}{N}$. The data from public health science data center showed that the new infected HBV case is 18725 in 2004. Hence, we estimate that the initial value of *M*(*t*) is *M*(0) = 18725. The other initial conditions are assumed to be *S*(0) = 3000000, *A*(0) = 30000, *C*(0) = 1960000 and *R*(0) = 9000000 respectively.
- The parameter *β*_2_ is obtained by fitting the model to data. By the least-square estimation, the transmission coefficient is estimated as *β*_2_ = 0.08078 and the 95% bootstrap confidence interval of *β*_2_ is (0.07229,0.09025).

**Table 2:** Parameters and their values.

Parameters	Value	Unit	Source
*ω*	0.48	year^−1^	[12]
*b*	0.0315	year^−1^	Fitting
*d*	0.0141	year^−1^	[26]
1 − *q*	0.11	year^−1^	[12]
*η*	0.885	year^−1^	[12]
*γ*	4	year^−1^	[12]
*α*	0.2%	year^−1^	[2]
*β*_2_	0.08078	year^−1^	Fitting

**Table 3:** The demographic data from 2004 to 2012 for Xinjiang(unit: ten thousand).

Year	2003	2004	2005	2006	2007
Population	1933.95	1963.11	2010.35	2050.00	2095.19
Year	2008	2009	2010	2011	2012
Population	2130.81	2158.63	2181.58	2208.71	2232.78

## Results

### Fitting results

Prediction accuracy is an important criterion for evaluating forecasting validity. For such a reason, an error analysis based on two statistical measure, i.e. the mean absolute percentage error (MAPE) and the root mean square percentage error (RMSPE), is employed to estimate model performances and reliability. The MAPE and the RMSPE are defined as

$$\text{MAPE}=(\frac{1}{n}\sum_{k=2}^{n}|\frac{x^{\left(0\right)}\left(k\right)-{\hat{x}}^{\left(0\right)}\left(k\right)}{x^{\left(0\right)}\left(k\right)}|)\times 100\%,$$

$$\text{RMSPE}=\sqrt{\frac{\sum_{k=2}^{n}{\left[\left(x^{\left(0\right)}\left(k\right)-{\hat{x}}^{\left(0\right)}\left(k\right)\right)/x^{\left(0\right)}\left(k\right)\right]}^{2}}{n-1}}\times 100\%,$$

where *x* ^(0)^(*k*) is the actual value at time *k*, ${\hat{x}}^{\left(0\right)}\left(k\right)$ is its fitting value and *n* is the number of data used for prediction. The criteria of MAPE and RMSPE are shown in Table 4(see [27, 28]).

**Table 4:** Criteria of MAPE and RMSPE.

MAPE and RMSPE	Forecasting Power
< 10%	Highly accurate forecasting
10–20%	Good forecasting
20–50%	Resonable forecasting
> 50%	Inaccurate forecasting

**Fig 4:**
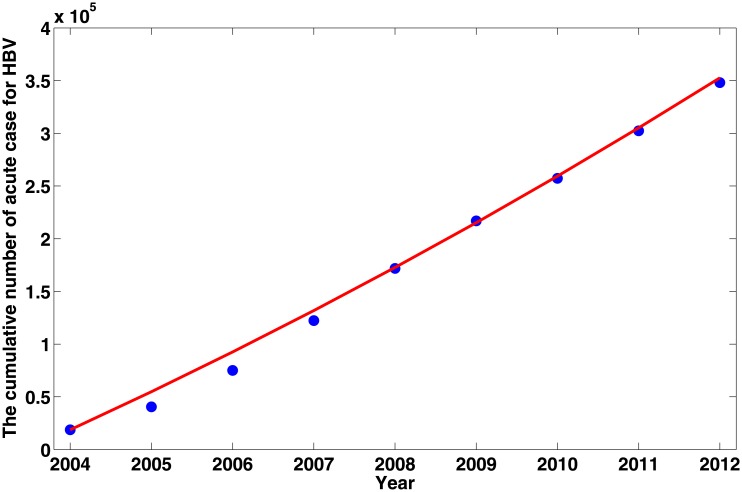
The comparison between the cumulative number of acute HBV case from 2004 to 2012 and the simulation of our model. The discrete points represent the data reported by public health science data center while the solid curve is simulated by using our model.

**Fig 5:**
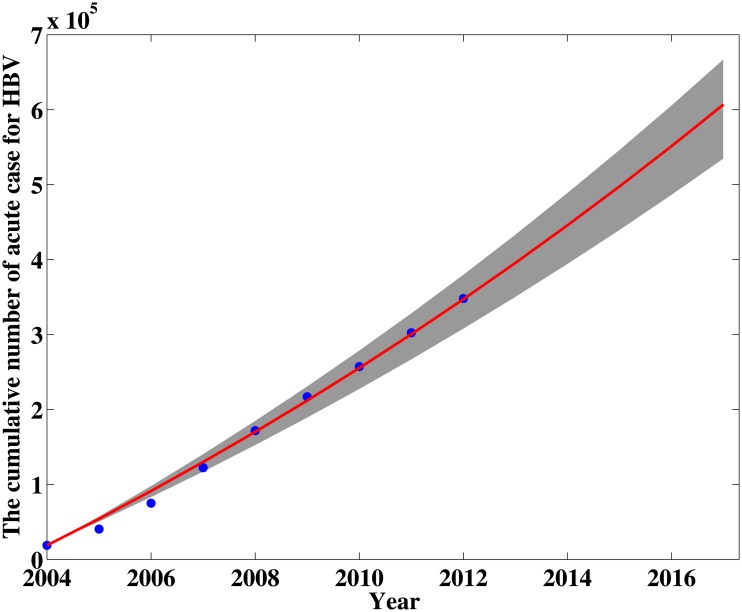
The tendency of the cumulative number of acute HBV cases from 2004 to 2017. Shaded areas represent 95% confidence interval around model predictions.

Based on the model and the parameter values in Table 2, we estimate the modified reproductive number to be *σ* = 1.1336(95% CI: 1.0144–1.2665). This indicates that the disease is uniform persistence. Therefore, if no further effective prevention and control measures are taken, the disease will not vanish. We can predict the general tendency of the epidemic according to the current situation(see Fig 4), which is presented in Fig 5, where MAPE = 7.70% and RMSPE = 15.26%.

### Sensitivity analysis

For the sensitivity analysis, Latin hypercube sampling was used to sample parameters that appear in the derived expression for *σ* [29]. Uncertainty and sensitivity analysis based on Latin hypercube sampling has been previously applied to disease transmission models [30–32]. Thus, in order to examine the sensitivity of our results to parameter variations, we use Latin hypercube sampling to examine the dependence on the modified reproductive number *σ*.

**Fig 6:**
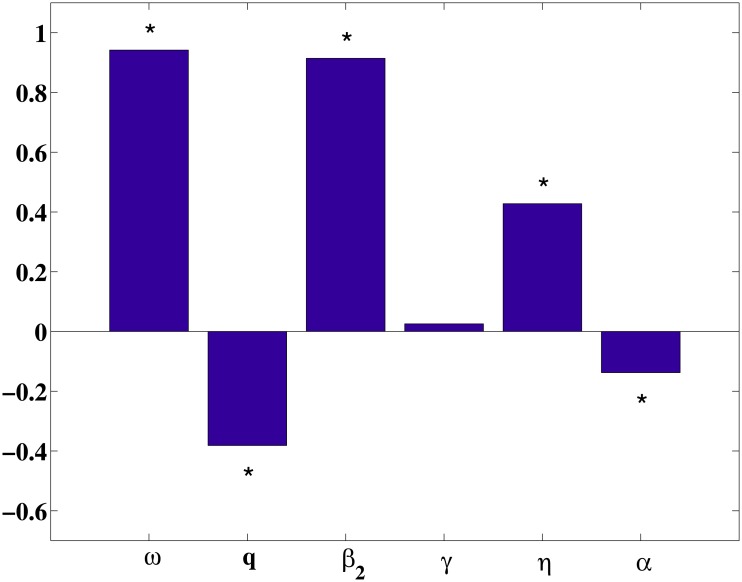
Partial rank correlation coefficients(PRCC) results for the dependence of *σ* on each parameter. * denotes the value of PRCC is not zero significantly, where the significance level is 0.05.

We choose the sample size *n* = 2000, parameters interested as the input variables, and the value of *σ* as the output variable. The PRCC values of six parameters are listed in Table 5 and shown on Fig 6. The ordering of these PRCCs corresponds to the level of statistical influence the parameter has on the variability for the modified reproductive number *σ*. The larger PRCCs in absolute value, the more important the parameter in responding to the change in *σ*. Plus sign or minus sign means the influence is positive or negative respectively. Fig 6 shows that *ω*, *β*_2_ and *η* have positive impact upon *σ*, whilst *q* and *α* have negative impact. We also know that *σ* is not sensitive to parameter *γ*. Further, Table 5 shows that unsuccessfully immunized proportion of newborns *ω*(∣PRCC∣ = 0.9418) has the greatest impact on *σ* followed by the transmission coefficient from carriers to susceptible individuals *β*_2_(∣PRCC∣ = 0.9143), then the proportion of leaving acute infection and progressing to carrier *η*. Hence, from sensitivity and mathematical analysis we conclude that the most effective approach to reduce the HBV infection is to decrease the parameters *ω* and *β*_2_.

**Table 5:** Partial rank correlation coefficients(PRCCs) for the aggregate *σ* and each input parameter variable.

Input parameter	The modified reproductive number *σ*	
	PRCC	*p* value
*ω*	0.9418	0
*β*_2_	0.9143	0
*η*	0.4277	1.1511 × 10^−67^
*q*	-0.3815	5.5550 × 10^−53^
*α*	-0.1375	9.4452 × 10^−8^
*γ*	0.0254	0.3263

**Fig 7:**
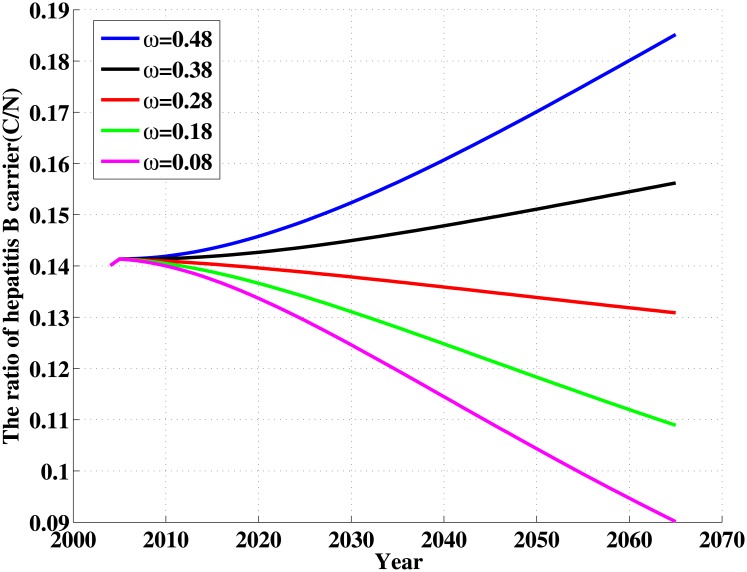
The influence of the parameter *ω* on the ratio of hepatitis B carrier ($\frac{C}{N}$). $\frac{C}{N}$ in terms of different values of ω.

**Fig 8:**
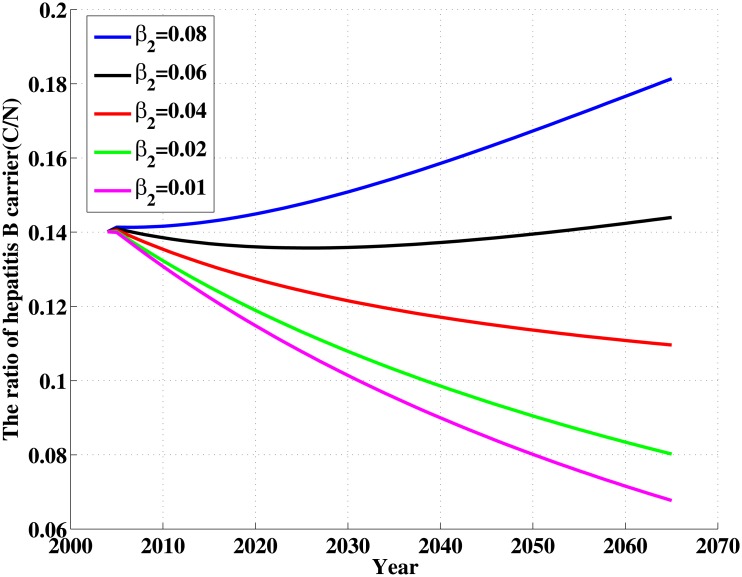
The influence of the parameter *β*_2_ on the ratio of hepatitis B carrier ($\frac{C}{N}$). $\frac{C}{N}$ in terms of different values of *β*_2_.

In the following, we focus on parameters *ω* and *β*_2_. The influence of the parameters *ω* and *β*_2_ on the ratio of hepatitis B carrier is shown in Figs 7 and 8. We can see from Fig 7 that when *ω* = 0.08, that is, proportion of newborns that is successfully immunized reaches 92%, the ratio of hepatitis B carrier will drop to 9% in 2065. When transmission coefficient *β*_2_ is reduced to 0.01, which is about 1/8 of current level, the ratio of hepatitis B carrier will drop to 8% in 2065(see Fig 8). This shows that the decline for the ratio of hepatitis B carrier will be a slow process. The most important factors lead to this phenomenon are the difficulty for curing the disease and long-term survival for carriers. Therefore, control and elimination of hepatitis is a long-term and arduous campaign.

## Discussion

Hepatitis B virus infection exists widely everywhere in the world. In Xinjiang, China, it is a high-prevalence infectious disease. In this paper, we have considered a hepatitis B model to explore the transmission dynamics and to give a prediction for the HBV infection trend in Xinjiang, China. Unlike the previous hepatitis models [2, 12, 13, 15, 16], the birth rate function in our model is not a constant but an exponential form. Thus, solutions of this model may be “blow up”. For solving this problem, we present a normalization to this model. The modified reproductive number is obtained which can determine whether the disease is extinct or not.

The simulation results can reflect the main trend of HBV epidemic in Xinjiang and also can give a prediction for the HBV infection trend. Fig 4 shows the yearly estimated cumulate HBV cases are very close to the data from 2004 to 2012. The prediction (see Fig 5) shows that the cumulative cases will continue increasing and attain about 600,000 by the end of 2017 unless there are stronger prevention measures. Furthermore, the model in this paper can only be used to fit short period HBV infection in Xinjiang, but not to continue the simulation to make predictions for the future. An important reason is preventing and controlling measures in Xinjiang have been adopted year by year. these measures will influence the parameters of the model is quantitatively impossible to predict. The sensitivity coefficients (PRCC) of the parameters with respect to the modified reproductive number *σ* are shown in Fig 6. The result indicates the unsuccessfully immunized proportion of newborns *ω* and the effective contact rate *β*_2_ between susceptible individuals and chronic HBV carriers are more sensitive to *σ* than the other parameters(see Figs 7 and 8). So, vaccination is also an important tool for controlling HBV infection. Particularly, vaccination policy for the newborns in Xinjiang need to be further strengthened. We suggest vaccination strategy at birth should cover all infants in Xinjiang. On the other hand, controlling contacts with hepatitis B patients is also an important measures to prevent transmission and spread of hepatitis B, especially with chronic patients. Here, we need to take effective measures to decrease not only directly but also indirectly contacts with HBV. Moreover, all the other parameters, such as the vertical transmission rate, the changing rate from acute to chronic stage etc., are all sensitive to *σ*. It tell us other effective controlling measures, for example prevention of mother-to-child transmission programmes, all susceptible population vaccination etc., are also beneficial to decreasing the prevalence of HBV in Xinjiang.

## Supporting Information

S1 Supporting Information Stability of the disease-free equilibrium and uniform persistence of system (5) are given in this file. (PDF)
