# Supplementary material for: Modeling and Analyzing the Transmission Dynamics of HBV Epidemic in Xinjiang, China
Source: PLoS One. 2015 Sep 30;10(9):e0138765. doi: 10.1371/journal.pone.0138765 (PMC4589324; doi:10.1371/journal.pone.0138765)
Supplement: S1 Supporting Information — (PDF) [file pone.0138765.s001.pdf]

S1 Support Information

Modeling and analyzing the transmission dynamics of HBV epidemic in Xinjiang, China

Tailei Zhang, Kai Wang, and Xueliang Zhang

In this supplementary material, we provide more detailed analysis for dynamical behaviors of the HBV model, including the stability of the disease-free equilibrium and the permanence of disease.

Stability of the disease-free equilibrium

To study the dynamical behaviors, we discuss the system as follows.

$$\begin{cases} \frac{\mathrm{d}x}{\mathrm{d}t} = & \omega[b(1-z)+bqz] - \beta_1 xy - \beta_2 xz - (b+\theta p)x + \alpha xz, \\ \frac{\mathrm{d}y}{\mathrm{d}t} = & \beta_1 xy + \beta_2 xz - (b+\gamma)y + \alpha yz, \\ \frac{\mathrm{d}z}{\mathrm{d}t} = & \eta\gamma y - (bq+\alpha)z + \alpha z^2. \end{cases} \tag{1}$$

Theorem 1. *The disease-free equilibrium $P_0(\bar{x}, 0, 0)$ of (1) is locally asymptotically stable in Γ if $\sigma < 1$; it is unstable if $\sigma > 1$.*

Proof. We analyze the stability of the disease-free equilibrium by investigating the eigenvalues of the Jacobian matrix of model (1) at P_0. The matrix is

$$J(P_0) = \begin{pmatrix} -(b+\theta p) & -\beta_1\bar{x} & -\omega b(1-q) - \beta_2\bar{x} + \alpha\bar{x} \\ 0 & \beta_1\bar{x} - (b+\gamma) & \beta_2\bar{x} \\ 0 & \eta\gamma & -(bq+\alpha) \end{pmatrix}. \tag{2}$$

The characteristic equation of $J(P_0)$ is

$$(\lambda + b + \theta p) \begin{vmatrix} \lambda - \beta_1\bar{x} + (b+\gamma) & -\beta_x\bar{x} \\ -\eta\gamma & \lambda + (bq+\alpha) \end{vmatrix} = 0. \tag{3}$$

The characteristic equation (3) has three eigenvalues, where $\lambda_1 = -(b+\theta p) < 0$, λ_2 and λ_3 are determined by the equation as follows.

$$\lambda^2 + a_1\lambda + a_2 = 0, \tag{4}$$

in which

$$\begin{aligned} a_1 &= -\beta_1\bar{x} + b + \gamma + bq + \alpha, \\ a_2 &= -(\beta_1(bq+\alpha) + \beta_2\eta\gamma)\bar{x} + (b+\gamma)(bq+\alpha). \end{aligned}$$

When $\sigma < 1$, we have $a_1 > 0$ and $a_2 > 0$ which implies that both λ_2 and λ_3 have negative real parts. When $\sigma > 1$, $a_2 < 0$ which implies that one of λ_2 and λ_3 is a positive real root. Thus the disease-free equilibrium is locally asymptotically stable if $\sigma < 1$ and unstable if $\sigma > 1$. Thus, these results establish the theorem.

In the next part, we will discuss the global stability of the disease-free equilibrium. The following lemma will be used in the proof of global stability for the disease-free equilibrium (see [1], Lemma 3.2).

Lemma 1. Let $\Delta = \{(u, v) \in \mathbb{R}_+^2 : 0 \le u + v \le 1\}$ and

$$h(u, v) = (a_1 - b_1)u + (c_1 - b_1)v + b_1.$$

Then, for any positive constants a_1, b_1 and c_1,

$$\max_{(u,v)\in\Delta} h(u,v) = \max\{a_1, b_1, c_1\}.$$

Theorem 2. *If $\sigma_0 < 1$, then the disease-free equilibrium P_0 is globally asymptotically stable, where*

$$\sigma_0 = \frac{\beta_1(bq+\alpha) + \max\{\beta_2, \alpha\}\eta\gamma}{(b+\gamma)(bq+\alpha)}. \tag{5}$$

Proof. Let

$$V(t) = \eta\gamma y + (b+\gamma-\beta_1)z. \tag{6}$$

The time derivative of V along system (1) satisfies

$$\begin{aligned}\dot{V}(t) = &\ \eta\gamma[(\beta_1 y+\beta_2 z)x-(b+\gamma)y+\alpha yz]\\ &+(b+\gamma-\beta_1)[\eta\gamma y-(bq+\alpha)z+\alpha z^2]\\ \le &\ \eta\gamma[(\beta_1 y+\beta_2 z)(1-y-z)-(b+\gamma)y+\alpha yz]\\ &+(b+\gamma-\beta_1)[\eta\gamma y-(bq+\alpha)z+\alpha z^2]\\ \le &\ z[h(y,z)-(bq+\alpha)(b+\gamma-\beta_1)].\end{aligned} \tag{7}$$

Here,

$$h(y,z) = (\alpha\eta\gamma-\beta_2\eta\gamma)y + ((b+\gamma-\beta_1)\alpha-\beta_2\eta\gamma)z + \beta_2\eta\gamma. \tag{8}$$

Applying Lemma 1 to (7), we obtain from (6)

$$\begin{aligned}\dot{V}(t) &\le [\max\{\alpha\eta\gamma, \beta_2\eta\gamma, (b+\gamma-\beta_1)\alpha\} - (bq+\alpha)(b+\gamma-\beta_1)]z\\ &\le 0, \quad \text{as } \sigma_0 < 1\end{aligned}$$

At the same time, $\dot{V} = 0$ only if $z = 0$. Set

$$M = \{(x,y,z) \in \Gamma : z = 0\}.$$

The first two equations of (1) in the set M become

$$\begin{cases}\dfrac{dx}{dt} = \omega b - \beta_1 xy - (b+\theta p)x,\\[2mm] \dfrac{dy}{dt} = \beta_1 xy - (b+\gamma)y.\end{cases} \tag{9}$$

From the second equation of (9), we have $\dfrac{dy}{dt} \le [\beta_1 - (b+\gamma)]y$. Note $\sigma_0 < 1$ implies $\beta_1 - (b+\gamma) < 0$. Consequently, we have $y(t) \to 0$ as $t \to \infty$. Furthermore, the first equation of (9) implies $x(t) \to \bar{x}$ as $t \to \infty$. Therefore, the largest compact invariant set in M is the singleton $\{P_0\}$. LaSalle's Invariance Principle then implies that P_0 is globally stable.

Uniform persistence

In the next part, we will show that the disease is uniformly persistent when $\sigma > 1$. We say the disease is endemic if the infected fraction (including acute and chronic stage) of the population persists above a certain positive level for sufficiently large time. That is to say there exists a $c > 0$ such that

$$\liminf_{t\to\infty}(y(t)+z(t)) \ge c.$$

The following result shows that the disease will become an endemic under the meaning of persistence of the disease.

Theorem 3. *The disease of system (1) is uniform persistence in Γ if $\sigma > 1$.*

Proof. We will use the results of Thieme in Theorem 4.6 of [2] to prove it. Define

$$\begin{aligned} X = & \quad \{(x,y,z) : x \geq 0, y \geq 0, z \geq 0, x+y+z \leq 1\}, \\ X_0 = & \quad \{(x,y,z) \in X : y+z > 0\}, \\ \partial X_0 = & \quad X \setminus X_0. \end{aligned} \tag{10}$$

In the following, we will show that system (1) is uniformly persistent with respect to $(X_0, \partial X_0)$.

Obviously, both X and X_0 are positively invariant with respect to (1). Furthermore, there exists a compact set $\mathfrak{B}$ in which all solutions of (1) initiated in X will enter and remain forever after. The compactness condition $(C_{4.2})$ in Thieme (1993 [2]) is easily verified for this set $\mathfrak{B}$. Denote

$$M_\partial = \{(x,0,0) : x \geq 0\}. \tag{11}$$

Write

$$\Omega = \cup\{\omega(x(0),y(0),z(0)) : (x(0),y(0),z(0)) \in M_\partial\}.$$

Restricting (1) on M_∂ gives

$$\frac{\mathrm{d}x}{\mathrm{d}t} = \omega b - (b+\theta p)x. \tag{12}$$

It is easy to verify that system (12) has a unique equilibrium $\bar{x}$. Thus $(\bar{x},0,0)$ is the unique equilibrium of (1) in M_∂. It is easy to check that $\bar{x}$ is globally asymptotically stable. Therefore, we have $\Omega = \{P_0\}$. And P_0 is a covering of Ω, which is isolated and acyclic (since there exists no solution in M_∂ which links P_0 to itself). Finally, the proof will be done if we show P_0 is a weak repeller for X_0, i.e.

$$\limsup_{t\to\infty} \operatorname{dist}(\mathbf{\Phi}(t), P_0) > 0,$$

where $\mathbf{\Phi}(t) = (x(t),y(t),z(t))$ is an arbitrarily solution with initial value in X_0. By Leenheer and Smith's results (2003, Proof of Lemma 3.5, [3]), we only need to prove $W^s(P_0) \cap X_0 = \emptyset$ where $W^s(P_0)$ is the stable manifold of P_0. Suppose it is not true, then there exists a solution $(x(t),y(t),z(t))$ in X_0, such that

$$x(t) \to \bar{x}, y(t) \to 0, z(t) \to 0 \quad \text{as} \quad t \to \infty. \tag{13}$$

Since

$$\sigma = \frac{[\beta_1(bq+\alpha) + \beta_2\eta\gamma]\bar{x}}{(b+\gamma)(bq+\alpha)} > 1$$

which is equivalent to

$$\frac{\beta_2\bar{x}}{bq+\alpha} > \frac{b+\gamma-\beta_1\bar{x}}{\eta\gamma}.$$

Thus, we can choose $\varepsilon > 0$, $\rho_1 > 0$ and $\rho_2 > 0$ such that

$$\frac{\beta_2(\bar{x}-\varepsilon)}{bq+\alpha} > \frac{\rho_2}{\rho_1} > \frac{b+\gamma-\beta_1(\bar{x}-\varepsilon)}{\eta\gamma}. \tag{14}$$

For $\varepsilon > 0$, by (13) there exists a $T > 0$ such that

$$\bar{x}-\varepsilon < x(t) < \bar{x}+\varepsilon, 0 < y(t) < \varepsilon, 0 < z(t) < \varepsilon$$

for all $t \geq T$. Let

$$V(t) = \rho_1 y(t) + \rho_2 z(t).$$

The derivative of V along the solution $(x(t), y(t), z(t))$ is given by

$$\begin{aligned}\dot{V}(t) = & \rho_1[(\beta_1 y + \beta_2 z)x - (b+\gamma)y + \alpha yz] \\ & +\rho_2[\eta\gamma y - (bq+\alpha)z + \alpha z^2] \\ \geq & \rho_1[(\beta_1 y + \beta_2 z)(\bar{x} - \varepsilon) - (b+\gamma)y] \\ & +\rho_2[\eta\gamma y - (bq+\alpha)z] \\ = & [\rho_2\eta\gamma - \rho_1(b+\gamma-\beta_1(\bar{x}-\varepsilon))]y \\ & +[\rho_1\beta_2(\bar{x}-\varepsilon) - \rho_2(bq+\alpha)]z \\ \geq & \rho V(t)\end{aligned}$$

for all $t \geq T$, where (14) implies

$$\rho = \min\{\frac{\rho_2\eta\gamma - \rho_1(b+\gamma-\beta_1(\bar{x}-\varepsilon))}{\rho_1}, \frac{\rho_1\beta_2(\bar{x}-\varepsilon) - \rho_2(bq+\alpha)}{\rho_2}\} > 0.$$

Hence $V(t) \to \infty$ as $t \to \infty$, which contradicts to the boundedness of $V(t)$. This completes the proof of Theorem 3.

References

1. Li MY, Graef JR, Wang L, Karsai J. Global dynamics of a SEIR model with varying total population size. Mathematical biosciences. 1999;160(2):191–213.

2. Thieme HR. Persistence under relaxed point-dissipativity (with application to an endemic model). SIAM Journal on Mathematical Analysis. 1993;24(2):407–435.

3. Smith HL, De Leenheer P. Virus dynamics: a global analysis. SIAM Journal on Applied Mathematics. 2003;63(4):1313–1327.
